# A Case of Chronic Avulsion Fracture of the Lesser Tuberosity of the Humerus in an Adolescent

**DOI:** 10.7759/cureus.94209

**Published:** 2025-10-09

**Authors:** Suguru Mikami, Yohei Shimada, Shu Somemura, Koh Terauchi, Naoki Haraguchi

**Affiliations:** 1 Department of Orthopedic Surgery, St. Marianna University School of Medicine, Kawasaki, JPN

**Keywords:** adolescent, avulsion fracture, lesser tuberosity, non-union, shoulder injury suture anchor fixation

## Abstract

This case report presents a rare instance of chronic avulsion fracture of the humeral lesser tuberosity in a 14-year-old male athlete. Initially misdiagnosed as a contusion, the injury resulted in persistent pain and restricted shoulder function. Advanced imaging, including CT and MRI, identified a non-union fracture. Surgical management was undertaken through a deltopectoral approach, employing suture bridge fixation with suture anchors, which secured stable fixation and achieved bone union within four months. At the six-month postoperative mark, the patient demonstrated complete recovery of shoulder strength and range of motion, resuming athletic activities without complications. This case underscores the diagnostic complexities associated with such fractures in adolescents, particularly due to the limited sensitivity of standard radiographs. Early utilization of MRI or CT imaging, alongside a suitable surgical strategy, is essential for favorable outcomes in comparable cases. This report underscores the necessity for enhanced diagnostic precision and prompt intervention in the treatment of rare adolescent shoulder fractures.

## Introduction

Avulsion fractures of the lesser tuberosity of the humerus are relatively rare in children and adolescents, often occurring due to sports-related activities. Adolescents are at a higher risk of avulsion fractures due to their immature skeletal structure, with open epiphyseal plates concentrating the impact force [1]. The diagnosis of avulsion fractures of the lesser tuberosity in this age group presents significant challenges, and delayed diagnosis frequently results in the formation of non-unions [2]. Chronic non-unions can lead to persistent pain and functional impairment, making early diagnosis and timely intervention essential. Recent reviews on pediatric shoulder injuries have also emphasized the importance of recognizing these uncommon fracture patterns, highlighting the role of early imaging and accurate diagnosis in preventing chronic sequelae [2]. In this report, we present a case of an adolescent male with a delayed diagnosis and chronic avulsion fracture of the lesser tuberosity, treated surgically, and outline his postoperative progress.

## Case presentation

The patient was a 14-year-old male presenting with right shoulder pain. Six months earlier, he had fallen while playing basketball, impacting his right shoulder, which was initially diagnosed as a shoulder contusion based on radiographs alone by a local physician and managed conservatively. Due to persistent pain, he was referred to our institution. Physical examination revealed a range of motion (ROM) of 180° in elevation, 180° in abduction, 80° in external rotation, and internal rotation reaching the L1 level with the hand-behind-back, compared to the left (unaffected) side with 180° in elevation, 180° in abduction, 70° in external rotation, and internal rotation reaching the T6 level. Furthermore, the belly press test, bear hug test, and lift-off test were positive, indicating subscapularis muscle weakness.

Imaging findings

**Figure 1:**
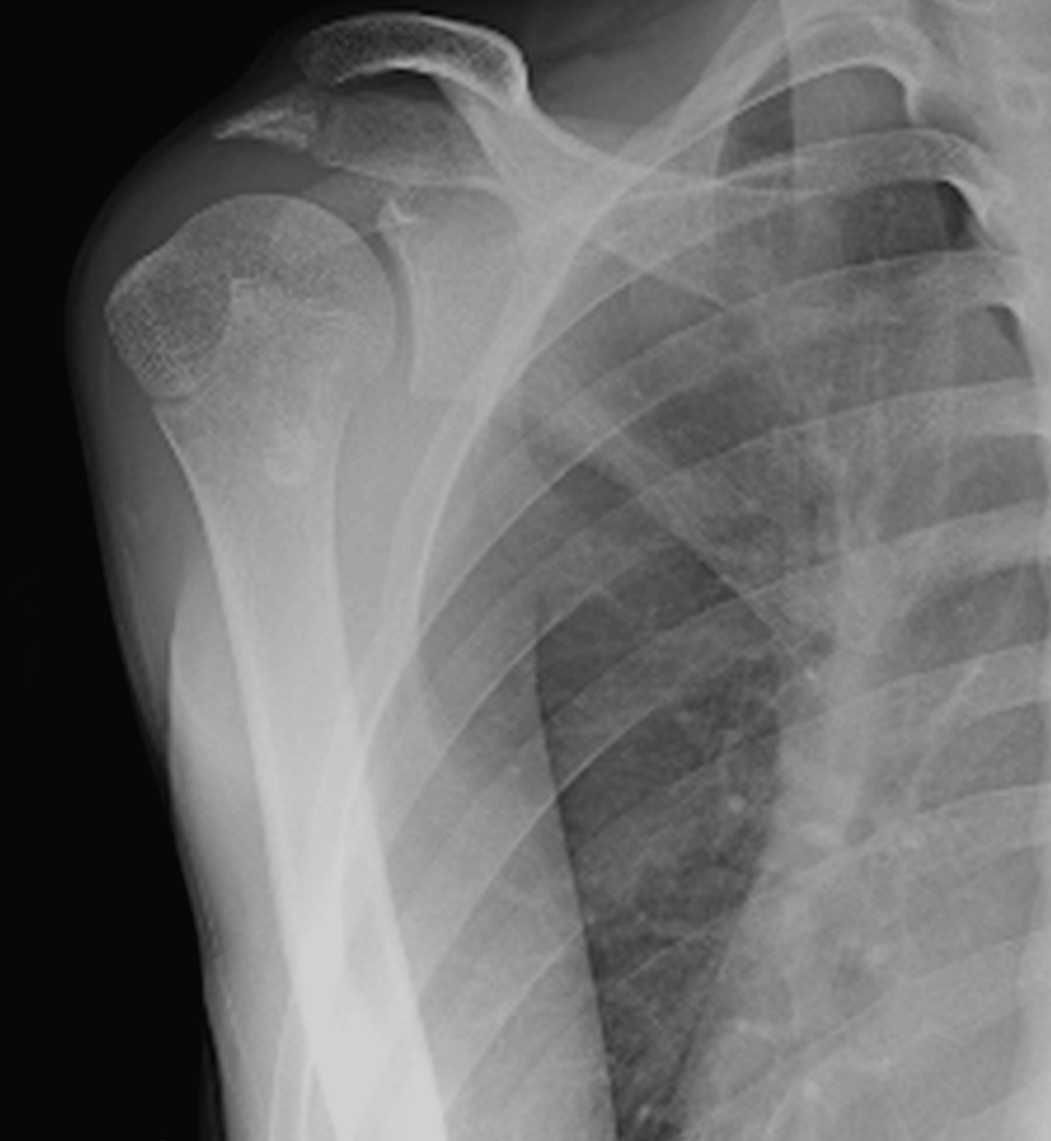
Plain radiography on the frontal view

**Figure 2:**
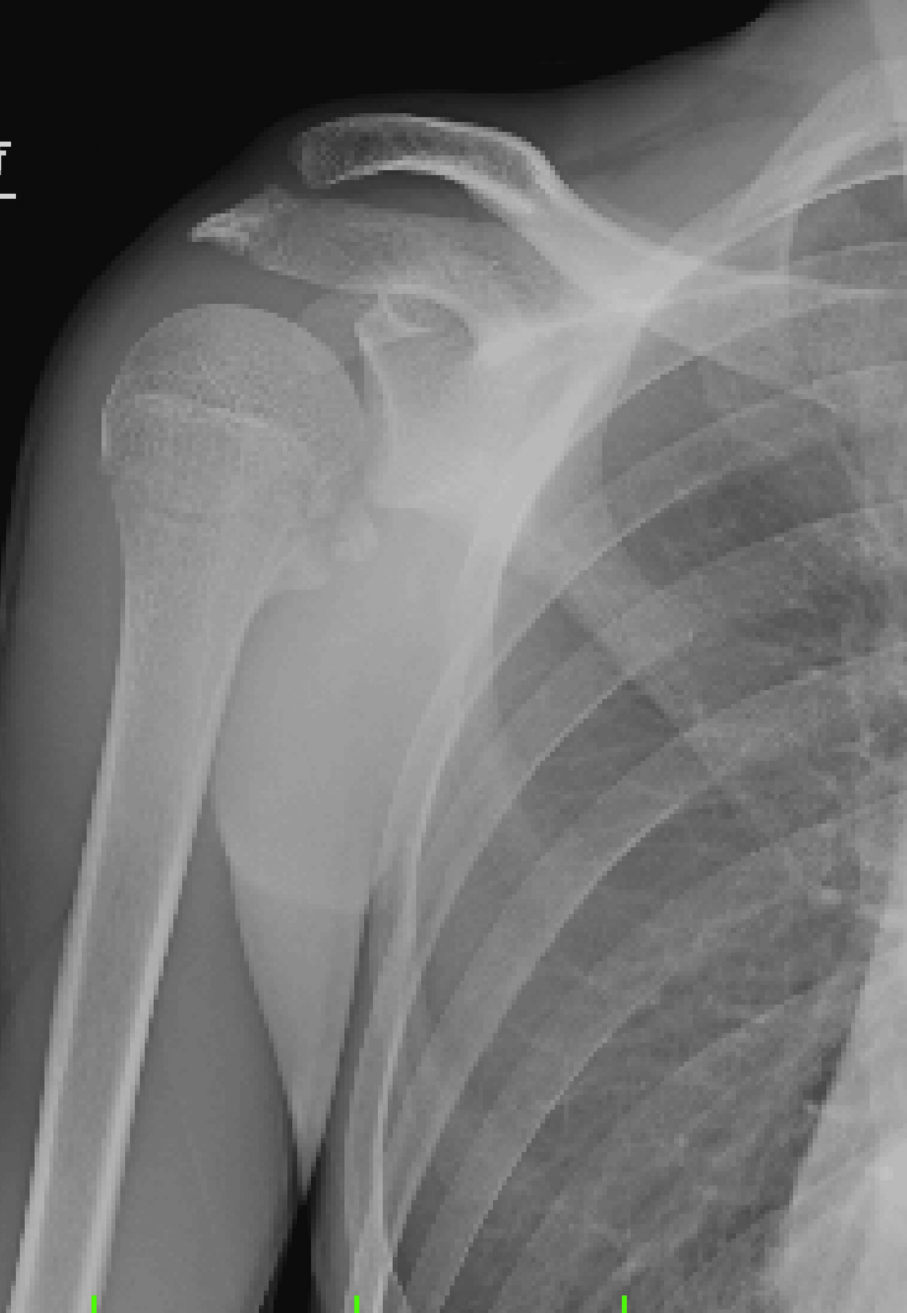
Plain radiography on the internal rotation view

Initial plain radiography did not reveal any abnormalities on the frontal view; however, an avulsed bone fragment was observed on the internal rotation view of the shoulder (Figures 1-2).

**Figure 3:**
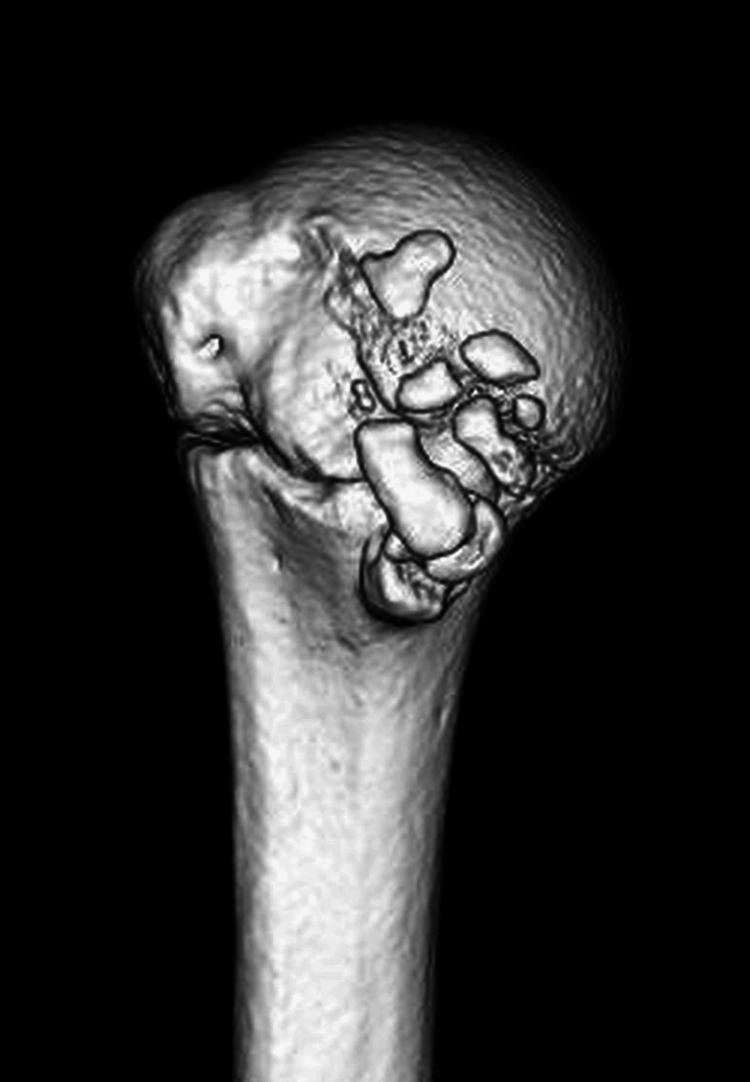
CT revealing multiple fragments at the lesser tuberosity CT: computed tomography

CT demonstrated multiple fragments at the lesser tuberosity, indicative of non-union (Figure 3).

MRI confirmed the presence of a bone fragment at the lesser tuberosity, with no evidence of subscapularis muscle tears or dislocation of the long head of the biceps tendon. Based on these findings, a diagnosis of chronic avulsion fracture of the lesser tuberosity was established. Persistent pain and the inability to resume basketball prompted the decision to proceed with surgical intervention. The patient and their family were informed that data from the research would be submitted for publication, and they provided their consent.

Surgical technique

**Figure 4:**
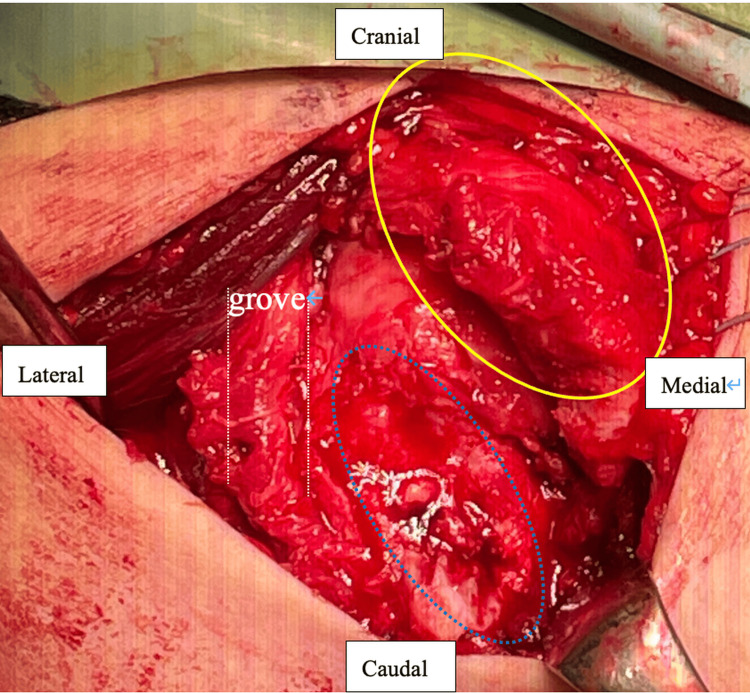
Intraoperative image showing the subscapularis muscle avulsed along with the lesser tuberosity (yellow circle) and detached from its insertion on the humeral rotator cuff footprint (blue arrow and white dotted lines)

A deltopectoral approach was employed, exposing the fracture site by separating the deltoid and pectoralis major muscles. Extensive scarring was present over the fracture site, and upon incision of the scar tissue, the fragment was found to be unstable, indicating non-union (Figure 4).

**Figure 5:**
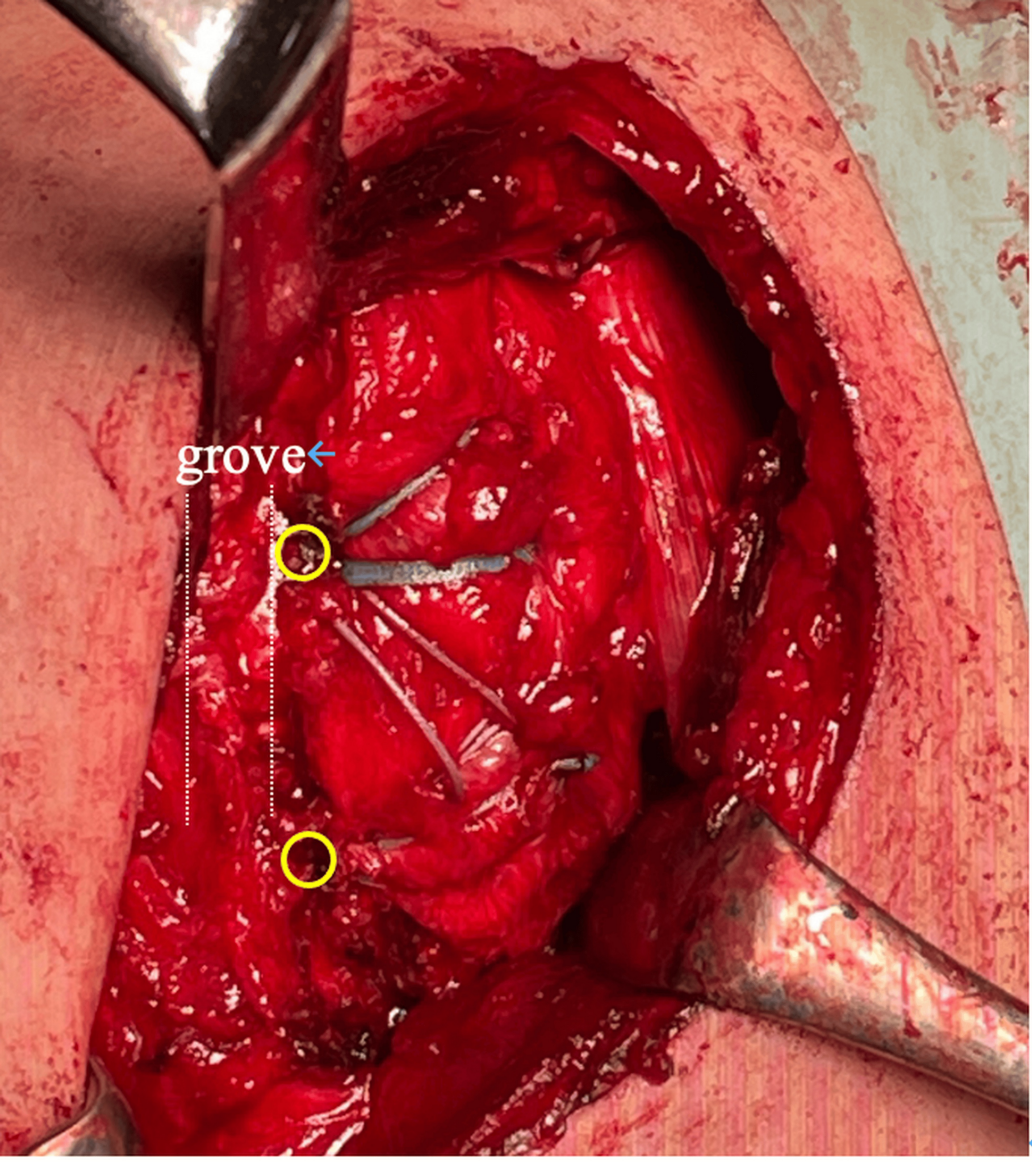
Bone fragment fixed with a suture bridge technique. The yellow circle represents the position of the lateral anchor

The subscapularis muscle was avulsed along with the bone fragment, and debridement of the lesser tuberosity footprint and the fragment was performed. Two suture anchors (Healix Advance™, 4.5 mm; DePuy Mitek, Raynham, MA, USA) were placed along the medial margin of the fracture to avoid physeal injury, and Mason-Allen sutures were applied to the subscapularis muscle. Following this, two lateral anchors (HAK®, 4.5 mm; Smith & Nephew, Andover, MA, USA) were positioned medial to the intertubercular groove, securing the bone fragment using a suture bridge technique (Figure 5).

Postoperative course

**Figure 6:**
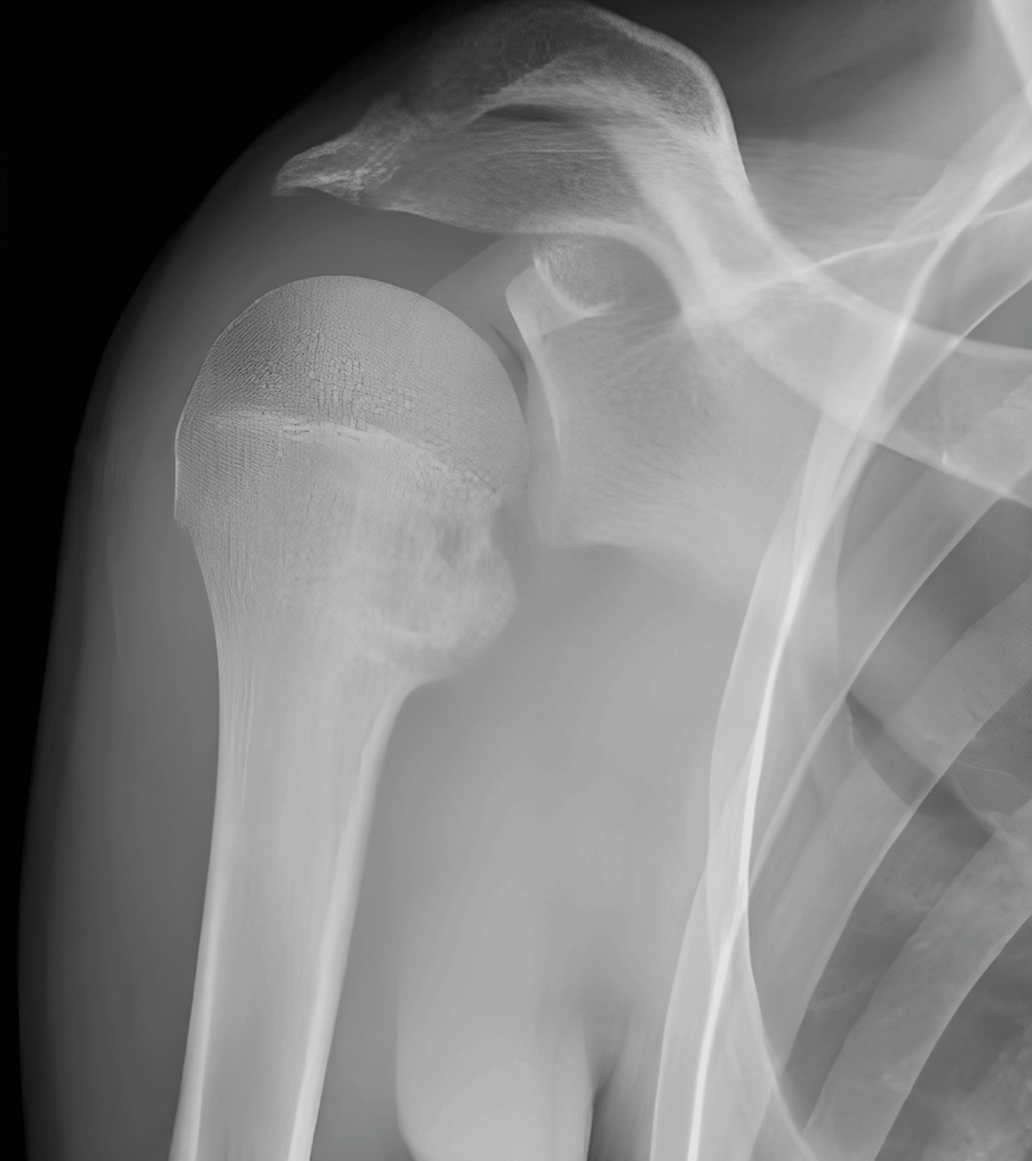
Plain radiography confirming bone union at four months postoperatively

The patient wore an abduction brace for four weeks, allowing passive and assisted ROM only in forward flexion (0-90°) and abduction (0-60°). No active internal rotation, pushing, lifting, or behind-the-back activities. Bone union was confirmed at four months postoperatively (Figure 6).

By six months, ROM in the right shoulder had improved to 180° in elevation, 180° in abduction, 70° in external rotation, and T8 in hand-behind-back internal rotation. Manual muscle tests for the subscapularis were all negative. The constant score improved from 78 preoperatively to 100 postoperatively, and he was able to resume playing basketball. One year post-surgery, the patient's progress has remained favorable without any complications.

## Discussion

Avulsion fractures of the lesser tuberosity in adolescents are classified as "transitional fractures" because they occur during a skeletal phase when the epiphyseal plate remains open [1]. They are commonly seen between the ages of 12 and 15 years [1].​ The mechanisms of injury typically involve eccentric contraction of the subscapularis or external rotation forces in extension [2,3]. In this case, the injury occurred during a basketball fall, where the shoulder was in extension, likely subjecting the subscapularis to significant strain.

CT or MRI plays a pivotal role in diagnosing these fractures because the sensitivity of plain radiographs is relatively low, at approximately 16%. In contrast, CT or MRI offers significantly higher sensitivity, around 95% [2]. The average delay in diagnosis is about two months, with cases exceeding six months not uncommon [2]. In cases with delayed diagnosis, non-union and chronic pain with functional limitations may occur, underscoring the need for early MRI or CT imaging.

Surgical methods, including arthroscopic and open surgery, yield favorable outcomes [2,4]; however, selecting the appropriate fixation method depends on the size and fragmentation of the bone fragment. Screw fixation is suitable for large fragments, while suture anchors or transosseous sutures are preferred for smaller or comminuted fragments [4,5].​ Although suture anchors provide less fixation strength in cancellous bone and transosseous sutures carry the risk of injury to the long head of the biceps tendon [4,5], the suture bridge technique using suture anchors was effective in this case of small fragments. It facilitated stable fixation, promoting early bone healing and enabling a timely return to sports activity.

This report describes a single case with follow-up limited to one year. Therefore, the outcomes presented here cannot be generalized, and further studies with larger cohorts and longer follow-up are needed to validate these findings.

## Conclusions

We report a rare case of chronic avulsion fracture of the lesser tuberosity of the humerus in an adolescent. This case highlights that, despite delayed diagnosis and non-union formation, favorable outcomes can be achieved through proper diagnosis and surgical treatment. Combining physical examination with MRI and CT imaging is essential for early and accurate diagnosis, and using the suture bridge technique with suture anchors proved effective for fixation. Improving diagnostic accuracy and optimizing surgical techniques will enhance outcomes in such rare cases.
